# Poly[μ_2_-aqua-aqua­(μ_3_-1*H*-benzimidazole-5,6-dicarboxyl­ato-κ^3^ *N* ^3^:*O* ^5^:*O* ^5′^)manganese(II)]

**DOI:** 10.1107/S1600536810011979

**Published:** 2010-04-10

**Authors:** Tan Zheng-De, Hai Qian-Qian, Yi Bing

**Affiliations:** a College of Chemistry and Chemical Engineering, Hunan Institute of Engineering, Xiang Tan 411104, People’s Republic of China

## Abstract

In the title complex, [Mn(C_9_H_4_N_2_O_4_)(H_2_O)_2_]_*n*_, the Mn^II^ atom is in a distorted octa­hedral coordination completed by one N atom from one 1*H*-benzimidazole-5,6-dicarboxyl­ate ligand, two O atoms from two different 1*H*-benzimidazole-5,6-dicarboxyl­ate ligands, and three O atoms from three water mol­ecules. Two bridging water mol­ecules and two bridging carboxyl­ate groups from a 1*H*-benzimidazole-5,6-dicarboxyl­ate ligand connect two Mn^II^ ions into a dimeric structure. In the crystal, extensive inter­molecular O—H⋯O, N—H⋯O and C—H⋯O hydrogen bonding forms a three-dimensional network.

## Related literature

For background to 1*H*-benzimidazole-5,6-dicarboxyl­ate complexes and related structures, see: Yao *et al.* (2008 ▶); Wei *et al.* (2009 ▶); Song *et al.* (2009*a* ▶,*b* ▶).
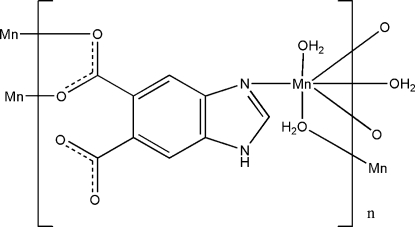


## Experimental

### 

#### Crystal data

[Mn(C_9_H_4_N_2_O_4_)(H_2_O)_2_]
*M* *_r_* = 295.11
Monoclinic, 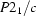

*a* = 8.8875 (18) Å
*b* = 9.2079 (18) Å
*c* = 12.939 (3) Å
β = 97.22 (3)°
*V* = 1050.5 (4) Å^3^
*Z* = 4
Mo *K*α radiation
μ = 1.28 mm^−1^
*T* = 293 K
0.29 × 0.26 × 0.25 mm

#### Data collection

Rigaku/MSC Mercury CCD diffractometer
Absorption correction: multi-scan (*REQAB*; Jacobson, 1998 ▶) *T*_min_ = 0.708, *T*_max_ = 0.740
8122 measured reflections
1888 independent reflections
1792 reflections with *I* > 2σ(*I*)
*R*_int_ = 0.025

#### Refinement

*R*[*F* ^2^ > 2σ(*F* ^2^)] = 0.036
*wR*(*F* ^2^) = 0.098
*S* = 1.11
1888 reflections
163 parameters
18 restraints
H-atom parameters constrained
Δρ_max_ = 0.71 e Å^−3^
Δρ_min_ = −0.84 e Å^−3^

### 

Data collection: *RAPID-AUTO* (Rigaku, 1998 ▶); cell refinement: *RAPID-AUTO*; data reduction: *CrystalStructure* (Rigaku/MSC, 2002 ▶); program(s) used to solve structure: *SHELXS97* (Sheldrick, 2008 ▶); program(s) used to refine structure: *SHELXL97* (Sheldrick, 2008 ▶); molecular graphics: *ORTEPII* (Johnson, 1976 ▶); software used to prepare material for publication: *SHELXL97*.

## Supplementary Material

Crystal structure: contains datablocks I, global. DOI: 10.1107/S1600536810011979/zb2004sup1.cif

Structure factors: contains datablocks I. DOI: 10.1107/S1600536810011979/zb2004Isup2.hkl

Additional supplementary materials:  crystallographic information; 3D view; checkCIF report

## Figures and Tables

**Table 1:** Hydrogen-bond geometry (Å, °)

*D*—H⋯*A*	*D*—H	H⋯*A*	*D*⋯*A*	*D*—H⋯*A*
N2—H2⋯O2^i^	0.86	1.98	2.839 (3)	174
O2*W*—H4*W*⋯O3^ii^	0.84	2.56	3.065 (3)	120
O1*W*—H1*W*⋯O2^ii^	0.84	2.10	2.819 (3)	143
O2*W*—H3*W*⋯O1*W*^iii^	0.84	2.10	2.934 (3)	169
O1*W*—H2*W*⋯O1^iv^	0.84	1.77	2.575 (3)	160
C4—H4⋯O4^v^	0.93	2.48	3.216 (3)	136

Symmetry codes: (i) 
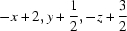
; (ii) 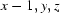; (iii) 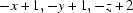; (iv) 
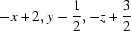
; (v) 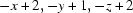.

**Fig. 1.**
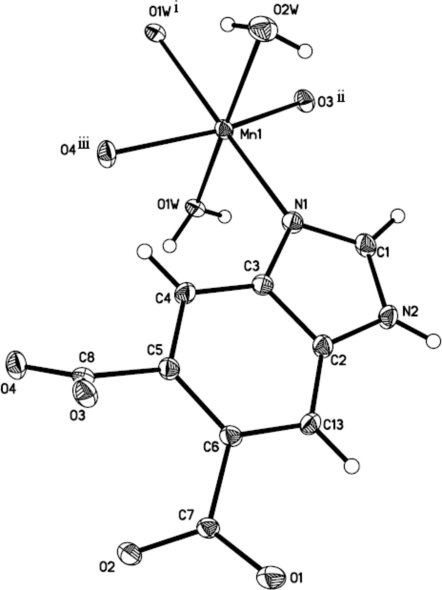
A section of the structure of the title compound, showing the atomic numbering scheme with 30% probability displacement ellipsoids. [Symmetry codes: (i) 1-x, 1-y, 1-z. (ii)y, x-1, z. (iii) 2-x, 1-y,2-z.]

**Fig. 2.**
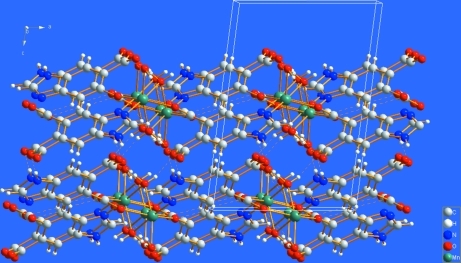
A view of the three-dimensional structure of the title compound along the b axis.Hydrogen bonds are shown as dashed lines.

## supplementary crystallographic information

### Comment

1*H*-Benzimidazole-5,6-dicarboxylate ligand(H_2_L) play an important role in coordination chemistry. They usually adopt diverse binding modes as monodentate, chelating to one metal center, bridging to two metal centers (Yao *et al.*, 2008;Wei *et al.*, 2009;Song *et al.*, 2009a,b). In the present paper, we synthesized a novel colorless complex [Mn(C_9_H_4_N_2_O_4_)(H_2_O)_2_]_n_. It is isostructural to the cobalt compound with reference of Wei *et al.*, 2009.

The coordination geometries of Mn centers are very close to the values observed in the [Co(C_9_H_4_N_2_O_4_)(H_2_O)_2_]_n_ compound. The Mn atoms are linked by water bridges and carboxylate groups, forming an infinite chain. The Mn···Mn distance is 3.2555 (7)Å longer than the Co···Co distance 3.114 (1) Å. In the [Co(C_9_H_4_N_2_O_4_)(H_2_O)_2_]_n_ compound, the Co—O bond lengths range between 2.0304 (18) and 2.2314 (19) Å, whereas in the title compound, the Mn—O bond lengths ranged between 2.1151 (19) and 2.3334 (19) Å. Intermolecular O—H···O , N—H···O and C—H···O hydrogen bonds form the 3D structure (Fig. 2). The hydrogen bonds are in the normal range (Table 1).

### Experimental

MnCl_2_(0.1 mmol), H_2_L(0.1 mmol), H_2_O (15 ml) and a small amount NaOH for adjusting pH to 7 was placed in a 23 ml Teflon reactor,which was heated to 426 K for two days and then cooled to room temperature , and left to stand at room temperature for a few days, then the colorless block crystals were obtained.

### Refinement

Carbon and nitrogen bound H atoms were placed at calculated positions and were treated as riding on the parent C or N atoms with C—H = 0.93 Å, N—H = 0.86 Å, and with *U*_iso_(H) = 1.2 *U*_eq_(C, N). The water H-atoms were located in a difference map, and were refined with a distance restraint of O—H = 0.84 Å; their *U*_iso_ values were refined.

### Crystal data

**Table:**

[Mn(C_9_H_4_N_2_O_4_)(H_2_O)_2_]	*F*(000) = 596
*M**_r_* = 295.11	*D*_x_ = 1.866 Mg m^−^^3^
Monoclinic, *P*2_1_/*c*	Mo *K*α radiation, λ = 0.71073 Å
Hall symbol: -P 2ybc	Cell parameters from 9020 reflections
*a* = 8.8875 (18) Å	θ = 3.2–27.5°
*b* = 9.2079 (18) Å	µ = 1.28 mm^−^^1^
*c* = 12.939 (3) Å	*T* = 293 K
β = 97.22 (3)°	Block, colorless
*V* = 1050.5 (4) Å^3^	0.29 × 0.26 × 0.25 mm
*Z* = 4	

### Data collection

**Table:**

Rigaku/MSC Mercury CCD diffractometer	1888 independent reflections
Radiation source: fine-focus sealed tube	1792 reflections with *I* > 2σ(*I*)
graphite	*R*_int_ = 0.025
ω scans	θ_max_ = 25.2°, θ_min_ = 3.2°
Absorption correction: multi-scan (REQAB; Jacobson, 1998)	*h* = −10→10
*T*_min_ = 0.708, *T*_max_ = 0.740	*k* = −11→11
8122 measured reflections	*l* = −15→15

### Refinement

**Table:**

Refinement on *F*^2^	Primary atom site location: structure-invariant direct methods
Least-squares matrix: full	Secondary atom site location: difference Fourier map
*R*[*F*^2^ > 2σ(*F*^2^)] = 0.036	Hydrogen site location: inferred from neighbouring sites
*wR*(*F*^2^) = 0.098	H-atom parameters constrained
*S* = 1.11	*w* = 1/[σ^2^(*F*_o_^2^) + (0.0562*P*)^2^ + 1.2161*P*] where *P* = (*F*_o_^2^ + 2*F*_c_^2^)/3
1888 reflections	(Δ/σ)_max_ = 0.001
163 parameters	Δρ_max_ = 0.71 e Å^−^^3^
18 restraints	Δρ_min_ = −0.84 e Å^−^^3^

### Special details

**Table:**

Geometry. All esds (except the esd in the dihedral angle between two l.s. planes) are estimated using the full covariance matrix. The cell esds are taken into account individually in the estimation of esds in distances, angles and torsion angles; correlations between esds in cell parameters are only used when they are defined by crystal symmetry. An approximate (isotropic) treatment of cell esds is used for estimating esds involving l.s. planes.
Refinement. Refinement of *F*^2^ against ALL reflections. The weighted *R*-factor wR and goodness of fit *S* are based on *F*^2^, conventional *R*-factors *R* are based on *F*, with *F* set to zero for negative *F*^2^. The threshold expression of *F*^2^ > σ(*F*^2^) is used only for calculating *R*-factors(gt) etc. and is not relevant to the choice of reflections for refinement. *R*-factors based on *F*^2^ are statistically about twice as large as those based on *F*, and *R*- factors based on ALL data will be even larger.

### Fractional atomic coordinates and isotropic or equivalent isotropic displacement parameters (Å^2^)

**Table:**

	*x*	*y*	*z*	*U*_iso_*/*U*_eq_	
C1	0.6538 (3)	0.9382 (3)	0.9155 (2)	0.0248 (6)	
H1	0.5552	0.9678	0.9215	0.030*	
N1	0.7164 (2)	0.8221 (3)	0.96244 (19)	0.0229 (5)	
O1W	0.52018 (17)	0.5378 (2)	0.88035 (12)	0.0232 (4)	
Mn1	0.59756 (4)	0.64549 (4)	1.03462 (3)	0.01916 (18)	
C2	0.8810 (3)	0.9343 (3)	0.8676 (2)	0.0222 (6)	
N2	0.7455 (3)	1.0094 (3)	0.85830 (19)	0.0253 (5)	
H2	0.7241	1.0871	0.8226	0.030*	
O2	1.3361 (2)	0.7520 (2)	0.77220 (15)	0.0249 (4)	
O2W	0.6154 (2)	0.74283 (19)	1.18596 (14)	0.0364 (5)	
C3	0.8628 (3)	0.8183 (3)	0.9340 (2)	0.0204 (5)	
O3	1.3671 (2)	0.7185 (2)	0.99961 (16)	0.0247 (4)	
C4	0.9821 (3)	0.7226 (3)	0.9624 (2)	0.0229 (6)	
H4	0.9724	0.6463	1.0082	0.028*	
O4	1.2334 (2)	0.5175 (2)	0.95142 (17)	0.0281 (5)	
C5	1.1158 (3)	0.7451 (3)	0.9201 (2)	0.0204 (6)	
C6	1.1295 (3)	0.8575 (3)	0.8473 (2)	0.0208 (6)	
C7	1.2657 (3)	0.8663 (3)	0.7888 (2)	0.0194 (6)	
C8	1.2495 (3)	0.6528 (3)	0.9593 (2)	0.0211 (6)	
C13	1.01235 (18)	0.95590 (19)	0.82118 (13)	0.0237 (6)	
H13	1.0212	1.0321	0.7752	0.028*	
H4W	0.5700	0.8040	1.1451	0.028*	
H1W	0.4444	0.5657	0.8400	0.028*	
H3W	0.5794	0.6589	1.1754	0.028*	
H2W	0.5900	0.5089	0.8473	0.028*	
O1	1.2983 (3)	0.9885 (2)	0.75589 (18)	0.0341 (5)	

### Atomic displacement parameters (Å^2^)

**Table:**

	*U*^11^	*U*^22^	*U*^33^	*U*^12^	*U*^13^	*U*^23^
C1	0.0165 (13)	0.0247 (15)	0.0341 (15)	0.0037 (10)	0.0065 (11)	0.0005 (12)
N1	0.0160 (11)	0.0230 (12)	0.0309 (13)	0.0014 (9)	0.0079 (9)	0.0030 (10)
O1W	0.0217 (9)	0.0265 (10)	0.0230 (9)	0.0043 (8)	0.0091 (7)	0.0005 (8)
Mn1	0.0167 (3)	0.0175 (3)	0.0242 (3)	0.00077 (14)	0.00610 (17)	0.00014 (15)
C2	0.0182 (13)	0.0197 (13)	0.0291 (14)	0.0021 (10)	0.0048 (11)	0.0025 (11)
N2	0.0205 (12)	0.0214 (12)	0.0348 (13)	0.0057 (9)	0.0067 (10)	0.0086 (10)
O2	0.0248 (10)	0.0233 (10)	0.0285 (10)	0.0012 (8)	0.0102 (8)	−0.0021 (8)
O2W	0.0467 (14)	0.0317 (12)	0.0324 (12)	−0.0080 (10)	0.0109 (10)	−0.0085 (9)
C3	0.0183 (9)	0.0208 (9)	0.0225 (9)	0.0002 (8)	0.0047 (8)	0.0010 (8)
O3	0.0175 (10)	0.0255 (11)	0.0314 (10)	0.0030 (8)	0.0044 (8)	−0.0019 (8)
C4	0.0192 (13)	0.0208 (14)	0.0300 (14)	0.0011 (10)	0.0082 (11)	0.0071 (11)
O4	0.0191 (9)	0.0195 (10)	0.0470 (12)	0.0029 (7)	0.0089 (9)	0.0052 (9)
C5	0.0171 (13)	0.0184 (13)	0.0263 (14)	0.0001 (10)	0.0055 (10)	0.0010 (10)
C6	0.0182 (13)	0.0198 (14)	0.0256 (14)	−0.0015 (9)	0.0077 (11)	−0.0005 (10)
C7	0.0183 (13)	0.0180 (13)	0.0228 (13)	−0.0028 (9)	0.0068 (11)	−0.0012 (10)
C8	0.0187 (14)	0.0230 (15)	0.0239 (14)	0.0026 (10)	0.0112 (11)	0.0038 (10)
C13	0.0232 (14)	0.0189 (13)	0.0302 (14)	0.0006 (10)	0.0087 (11)	0.0066 (11)
O1	0.0359 (9)	0.0296 (9)	0.0411 (9)	−0.0028 (7)	0.0212 (7)	0.0013 (7)

### Geometric parameters (Å, °)

**Table:**

C1—N1	1.317 (4)	N2—H2	0.8600
C1—N2	1.339 (4)	O2—C7	1.257 (3)
C1—H1	0.9300	O2W—H4W	0.8407
N1—C3	1.396 (3)	O2W—H3W	0.8411
N1—Mn1	2.209 (2)	C3—C4	1.392 (4)
O1W—Mn1	2.2572 (18)	O3—C8	1.262 (4)
O1W—Mn1^i^	2.3334 (19)	O3—Mn1^iv^	2.1495 (19)
O1W—H1W	0.8386	C4—C5	1.384 (4)
O1W—H2W	0.8401	C4—H4	0.9300
Mn1—O4^ii^	2.115 (2)	O4—C8	1.256 (3)
Mn1—O2W	2.141 (2)	O4—Mn1^ii^	2.115 (2)
Mn1—O3^iii^	2.1496 (19)	C5—C6	1.414 (4)
Mn1—O1W^i^	2.3334 (19)	C5—C8	1.497 (4)
Mn1—H4W	2.0785	C6—C13	1.390 (3)
Mn1—H3W	1.8533	C6—C7	1.508 (4)
C2—N2	1.381 (3)	C7—O1	1.250 (3)
C2—C3	1.392 (4)	C13—H13	0.9300
C2—C13	1.393 (3)		
			
N1—C1—N2	113.6 (2)	N1—Mn1—H3W	118.2
N1—C1—H1	123.2	O1W—Mn1—H3W	147.6
N2—C1—H1	123.2	O1W^i^—Mn1—H3W	59.0
C1—N1—C3	104.6 (2)	H4W—Mn1—H3W	41.0
C1—N1—Mn1	126.53 (18)	N2—C2—C3	105.7 (2)
C3—N1—Mn1	127.15 (18)	N2—C2—C13	131.3 (2)
Mn1—O1W—Mn1^i^	90.32 (6)	C3—C2—C13	123.0 (2)
Mn1—O1W—H1W	123.2	C1—N2—C2	107.0 (2)
Mn1^i^—O1W—H1W	98.2	C1—N2—H2	126.5
Mn1—O1W—H2W	115.3	C2—N2—H2	126.5
Mn1^i^—O1W—H2W	114.5	Mn1—O2W—H4W	74.3
H1W—O1W—H2W	111.5	Mn1—O2W—H3W	58.9
O4^ii^—Mn1—O2W	104.41 (8)	H4W—O2W—H3W	111.8
O4^ii^—Mn1—O3^iii^	152.59 (8)	C2—C3—C4	120.3 (2)
O2W—Mn1—O3^iii^	91.19 (8)	C2—C3—N1	109.1 (2)
O4^ii^—Mn1—N1	100.75 (8)	C4—C3—N1	130.6 (3)
O2W—Mn1—N1	95.40 (9)	C8—O3—Mn1^iv^	130.88 (18)
O3^iii^—Mn1—N1	100.03 (8)	C5—C4—C3	117.6 (3)
O4^ii^—Mn1—O1W	84.15 (8)	C5—C4—H4	121.2
O2W—Mn1—O1W	166.37 (7)	C3—C4—H4	121.2
O3^iii^—Mn1—O1W	76.97 (7)	C8—O4—Mn1^ii^	128.63 (18)
N1—Mn1—O1W	93.33 (8)	C4—C5—C6	121.5 (2)
O4^ii^—Mn1—O1W^i^	78.58 (7)	C4—C5—C8	117.7 (2)
O2W—Mn1—O1W^i^	81.81 (7)	C6—C5—C8	120.6 (2)
O3^iii^—Mn1—O1W^i^	81.59 (7)	C13—C6—C5	121.0 (2)
N1—Mn1—O1W^i^	176.83 (8)	C13—C6—C7	117.8 (2)
O1W—Mn1—O1W^i^	89.68 (6)	C5—C6—C7	121.0 (2)
O4^ii^—Mn1—H4W	125.7	O1—C7—O2	123.6 (3)
O2W—Mn1—H4W	22.9	O1—C7—C6	117.1 (2)
O3^iii^—Mn1—H4W	74.5	O2—C7—C6	119.3 (2)
N1—Mn1—H4W	83.0	O4—C8—O3	126.1 (3)
O1W—Mn1—H4W	150.1	O4—C8—C5	117.2 (2)
O1W^i^—Mn1—H4W	94.9	O3—C8—C5	116.7 (2)
O4^ii^—Mn1—H3W	96.4	C6—C13—C2	116.4 (2)
O2W—Mn1—H3W	22.9	C6—C13—H13	121.8
O3^iii^—Mn1—H3W	89.3	C2—C13—H13	121.8

Symmetry codes: (i) −*x*+1, −*y*+1, −*z*+2; (ii) −*x*+2, −*y*+1, −*z*+2; (iii) *x*−1, *y*, *z*; (iv) *x*+1, *y*, *z*.

### Hydrogen-bond geometry (Å, °)

**Table:**

*D*—H···*A*	*D*—H	H···*A*	*D*···*A*	*D*—H···*A*
N2—H2···O2^v^	0.86	1.98	2.839 (3)	174
O2W—H4W···O3^iii^	0.84	2.56	3.065 (3)	120
O1W—H1W···O2^iii^	0.84	2.10	2.819 (3)	143
O2W—H3W···O1W^i^	0.84	2.10	2.934 (3)	169
O1W—H2W···O1^vi^	0.84	1.77	2.575 (3)	160
C4—H4···O4^ii^	0.93	2.48	3.216 (3)	136

Symmetry codes: (v) −*x*+2, *y*+1/2, −*z*+3/2; (iii) *x*−1, *y*, *z*; (i) −*x*+1, −*y*+1, −*z*+2; (vi) −*x*+2, *y*−1/2, −*z*+3/2; (ii) −*x*+2, −*y*+1, −*z*+2.
